# Survival and drifting patterns of grass carp eggs and larvae in response to interactions with flow and sediment in a laboratory flume

**DOI:** 10.1371/journal.pone.0208326

**Published:** 2018-12-19

**Authors:** Andres F. Prada, Amy E. George, Benjamin H. Stahlschmidt, Duane C. Chapman, Rafael O. Tinoco

**Affiliations:** 1 Department of Civil and Environmental Engineering, University of Illinois at Urbana-Champaign, Urbana, IL, United States of America; 2 U.S. Geological Survey, Columbia Environmental Research Center, Columbia, MO, United States of America; Brandenburgische Technische Universitat Cottbus-Senftenberg, GERMANY

## Abstract

A series of laboratory experiments was conducted to better understand the behavior of grass carp eggs and larvae in moving water in order to develop and implement new strategies for control and prediction of their dispersal and drift at early life stages. Settling velocity and density of a representative sample of eggs were estimated, and three trials of flume experiments with different flow conditions were conducted with live eggs in a temperature-controlled setting with a mobile sediment bed. In these trials, egg and larval stages were continuously analyzed over periods of 80 hours; and eggs and larvae interactions with the flow and sediment bed were monitored and characterized qualitatively and quantitatively. Survival rates were quantified after each trial, highlighting physical causes for increased mortality. Detailed flow analysis was correlated to the observed drifting and swimming behavior of eggs and larvae, to estimate distributions across the water depth, as well as traveling and swimming speeds. Evidence of the influence of mean and turbulent flow in the suspension and transport of eggs are reported, and swimming patterns of larvae at different developmental stages are described. These findings support the development of new strategies for monitoring the spread of grass carp eggs and larvae in rivers, and provide new inputs to predict conditions favorable for spawning and hatching, allowing for mitigation measures at early life stages, which are critical to control their dispersal.

## Introduction

Grass carp (*Ctenopharyngodon idella*) are native to eastern Asia, but they have been introduced and have become established in the central United States, where they are considered problematic invaders. In their native range, where wild populations are in severe decline [1], they are important culturally and economically as a food fish [2].

The primary ecological effect of the introduction of grass carp is reduction in aquatic macrophytes [3–5], which can be problematic where macrophytes are considered beneficial and where they serve as spawning locations and nursery habitat for native fishes. Grass carp have now invaded the Laurentian Great Lakes [6–7] where desirable macrophytes are already in decline and where grass carp are predicted to become a nuisance [3]. Forecasting the spread and effects of grass carp in the Great Lakes has become an area of intense concern and of research [3,8–11]. Several approaches to deter fish movements have been tested: electric barriers, strobe lights, acoustic fish deterrents, bubble curtains, velocity barriers, oxygen availability, pheromones, magnetic fields, and the use of chemicals ([12], and references therein).

Management measures at spawning times could also be adopted to prevent recruitment and to reduce population growth, by identifying survival and dispersal bottlenecks due to physical interactions during early life stages. After ecosystem invasion, it is difficult or impossible to eradicate them, thus prevention becomes a more effective mitigation tactic.

Grass carp and their relatives, the bigheaded carps (silver carp *Hypophthalmichthys molitrix* and big head carp *H*. *nobilis*) which are also considered highly undesirable invaders in the United States and highly beneficial in Asia, can spawn hundreds of thousands of eggs at a time in turbulent flows of large rivers and their tributaries. Eggs subsequently develop and hatch while drifting in the currents [13–15]. Therefore, understanding grass carp and bigheaded carp response to flow and turbulence regimes at early life stages is fundamental to monitoring and controlling their dispersal and drift.

Experiments on still water have found that egg survival is poor if they settle to the bottom and are buried in the sediment bed (e.g., [15–17]). Synthetic eggs have also been used in experiments to study egg transport under turbulent flow conditions, to evaluate suspension and settling dynamics of water-hardened eggs [18]. Numerical tools have been developed to simulate the drifting behavior of grass carp and bigheaded carps during their early life stages (e.g. FluEgg, [19]) and have been tested on rivers in Michigan and Illinois [20–21]. FluEgg incorporates the influence of flow velocity, shear velocity, and turbulent diffusion on the transport and dispersal patterns of grass carp and bigheaded carp eggs. However, it does not account for egg survival rates or larval behavior after hatching. Simulations end when eggs hatch, which is estimated based on the water temperature specified, and it does not inform mortality rates due to boundary interactions or the dispersal and drift of larvae. Physical experimentation is thus required to evaluate the influence of the river bed on survival rates and study the swimming behavior of larvae.

Recent laboratory experiments with live grass carp and bigheaded carp eggs [22] aimed to physically characterize eggs (i.e. estimate diameters, density, and terminal fall velocity) to better inform numerical models to estimate drifting. However, a comprehensive study that documents the hatching transition and the early behavior of larvae in moving water has not been conducted yet. A better understanding of the drifting of eggs and larvae under different flow and turbulence conditions, along with their interactions with a sediment bed would enhance model predictive capabilities for tracking the dispersal and drift of the early life stages of these invasive fishes.

In this study, three comprehensive trials of laboratory experiments were conducted in both still and moving water to determine the drifting and swimming behaviors of live grass carp eggs and larvae. Ranges of diameters, densities, and settling fall velocities of eggs were estimated in still water, whereas traveling speeds of eggs and larvae, as well as the swimming capabilities of larvae against the flow, were quantified in moving water. Continuous vertical distributions were also generated to observe the location and the transition from eggs to larvae across the water depth at different flow conditions; and survival rates were monitored at the end of each trial to ascertain possible causes of mortality based on both hydrodynamics and biological interactions.

## Materials and methods

The Illinois Department of Natural Resources granted us the authorization to possess diploid grass carp (*Ctenopharyngodon idella*) eggs and larvae in the laboratory facility for research purposes only. Measures were taken to prevent release of the fish from the facility, and waste water was disposed so as to prevent egg and larva escapement. MS-222 (tricaine methanesulfonate) was used to euthanize all surviving larvae. Remaining organic material was filtered and incinerated to prevent contamination and potential escapement of organic material. Experiments were conducted at the Ven Te Chow Hydrosystems Laboratory (VTCHL) at the University of Illinois in Urbana-Champaign. Fertilized grass carp eggs were cultured as described by George et al. [23], and transported from the U.S. Geological Survey (USGS) Columbia Environmental Research Center (CERC) in Columbia, MO. Three trials of experiments were conducted. During trial 1, two females (5.09 and 3.21 kg) and four males (2.81, 2.23, 2.79, 1.86 kg) were used for spawning. During trial 2, one female (6.53 kg) and four males (3.60, 3.37, 3.61, 3.24 kg) were used, and during trial 3, three females (4.76, 5.36, and 5.60 kg) and four males (4.76, 4.00, 4.40, 4.40 kg) were used.

George and Chapman 2015 [17] and George et al. 2017 [22] showed that there can be large variations in egg size, due to maternal effects (larger females producing larger eggs), and temperature effects, with eggs from cooler water being larger than eggs from warmer conditions. For the present study, eggs were purposely obtained from individuals of similar size amongst all trials, to account for the response to flow conditions using similar eggs. However, the wide range in sizes presented in the following sections indicate that the sizes studied are representative of most of the expected range of grass carp eggs in nature. Spawning on trials 1 and 3 resulted in batches of good quality eggs (well-defined membrane and well-formed fish inside). However, the batch for trial 2 was negatively affected by high water temperatures at spawning (temperatures over 24°C), resulting in poor quality eggs.

### Settling column test

**Fig 1:**
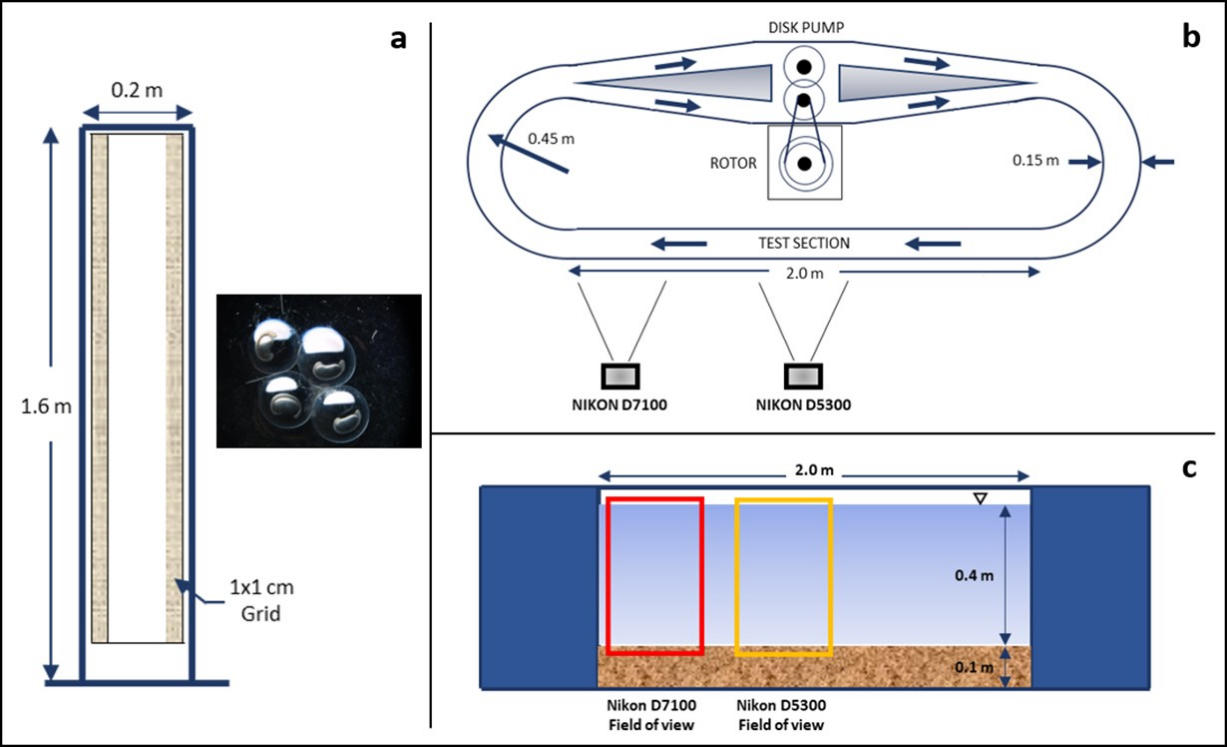
Facilities used to conduct the experiments (not to scale). (a) Settling water column with an inset of microscope image of the eggs. (b) Plan view of Race-Track Flume (RTF), Odell-Kovasznay type flume, with the setup of side-view cameras. (c) Side-view of the Race-Track Flume with the field of view of each camera.

Settling speeds (*w*_*s*_) of eggs were determined at different times after fertilization during trial 1 (at 6, 10, 14, 18, and 22 hours after fertilization) by taking photos of individual falling eggs (25 eggs at each time) at 1 frame every 3 seconds with a Nikon D200 camera (10.2MP) on a backlit rectangular settling column 1.6 m tall, 0.2 m long, and 0.1 m wide, with a 1x1cm vertical grid for spatial calibration (Fig 1A). A Nikon SMZ-1500 stereomicroscope was used to measure egg diameter prior to release, using the mean of two perpendicular measurements, with a tolerance of 0.01 mm (inset in Fig 1A).

Custom particle image processing MATLAB (MathWorks, Inc.) routines were developed to track the position of individual eggs and larvae in a series of consecutive frames. These routines produced binary images, which were processed using morphological processing tools to obtain the center of mass (*x*, *z* coordinates). Using such coordinates for each centroid and the difference in time between captures from video recordings, the traveling speeds between frames were calculated.

The slow settling speed of the eggs allowed the use of Stokes’ law to estimate the density of eggs (*ρ*_*s*_) over time as [24]:

$$\rho_{s}=\frac{18\mu w_{s}}{gd_{s}^{2}}+\rho$$ (1)

Where *ρ* and *μ* are the density and dynamic viscosity of water, and *d*_*s*_ is the egg diameter.

A one-way Analysis of Variance (ANOVA) was performed to test whether the settling speed of the eggs and their density changed over time before hatching. A significance level of α = 0.05 was considered statistically significant between the batches of eggs at the time intervals specified.

### Race-track flume test

Drifting behavior of eggs and larvae under different flow conditions, and their interaction with the sediment bed and turbulent structures, were monitored with cameras on each trial for ~80 consecutive hours in the RTF; an Odell-Kovasznay type flume [25], shown in Fig 1B and 1C. For trial 1 and trial 3, 4000 eggs were released on the flume, but for trial 2 only 1604 due to the poor-quality spawning.

Water depth in the RTF was maintained at *H =* 0.4 m, above a 0.1-m thick sediment bed, composed of a mixture of walnut shells and sand (bulk sediment density *ρ*_*p*_ = 1250 kg/m^3^, and size {D_16_, D_50_, D_84_} = {0.41, 0.54, 0.66} mm), placed only in the straight test section. All substrate material was washed and dried in an oven at 170 F (77°C) for 48 hours before each trial to help prevent biological contamination and control the spread of fungus. Settling velocity of the sediment was calculated as *w*_*p*_ = 1.89*x*10^-2^ m/s, using the simple universal equation for grain settling velocity presented by Ferguson and Church [26]:

$$w_{p}=\frac{Rgd_{p}^{2}}{C_{1}\nu +\sqrt{0.75C_{2}Rgd_{p}^{3}}}$$ (2)

where *R* is the submerged specific gravity $\left(R=\frac{\rho_{p}-\rho }{\rho }\right)$, *g* is the gravity, *d*_*p*_ is the nominal particle diameter (*D*_*50*_ was used), and *v* is kinematic viscosity of water, respectively. C_1_ and C_2_ are 20 and 1.1, respectively, based on shape factors associated with natural sediment [26].

The vertical distribution of eggs was captured by taking consecutive photos (1 per minute) during the first ~30 hours (Nikon D7100 camera, 24.1MP) over a straight test-section of the flume (Fig 1B and 1C). Egg trajectories and their interaction with a flat sediment bed were also recorded through 10-min videos every 4 hours at 60 fps (Nikon D5300, 24.2MP). After eggs hatched, the same analysis was conducted to evaluate swimming behavior of larvae until ~80 hours. The 0.40 m water depth in the images was divided into 9 equal intervals (0.044 m each) to count the number of eggs or larvae detected in each interval (1 minute). Nearly translucent eggs were visible in the images due to the opacity of the yolk. Once they hatched, approximately 27 hours after fertilization, larvae were almost transparent, hard to see with the naked eye, but identifiable against the lit background. At approximately 60 hours post fertilization, pigmentation in the eyes and back of the larvae increased, allowing for easier detection.

Vertical distributions were then plotted through the duration of the experiment, showing different patterns at various fish development periods. Three developmental periods were defined:

Period 1—embryonic stages; eggs prior to hatching (0 to 27 hours depending on the water temperature; developmental stages 8–30 [27]), which drift passively and settle in the absence of turbulence.
Period 2—after egg hatching and before gas bladder emergence (27 to 65 hours; stages 31–36 [27]); in laboratory studies, this stage is characterized by vertical swimming and falling [22].
Period 3—after gas bladder emergence (65 to 90 hours; stages 37–39 [27]). This period is characterized by horizontal swimming and the ability to maintain vertical position without swimming. Larvae of this period are often captured in off-channel low velocity habitats [22], which are often considered nursery areas for grass carp and, where development to older juvenile stages occurs.

In period 3, when fish began swimming horizontally, the horizontal swimming speed (*u*_*sw*_) was defined as:

$$u_{sw}=U-u_{t}$$ (3)

where *U* is the mean flow velocity and *u*_*t*_ is the horizontal traveling speed, obtained from the absolute displacement between consecutive frames and defined as positive in the direction of the flow. Swimming speed is thus defined as positive if the larva moves against the flow, and negative in the same direction of the flow.

#### Flow characterization in RTF

A different flow condition was applied in each trial. Flow is driven by a vertical-axis disk pump, controlled by a frequency inverter. A linear relationship exists between the assigned inverter frequency and the rotation speed of the disk pump, given by *Ω* [RPM] = 6.6 *f* [Hz]. For trial 1, a disk rotation rate of *f* = 10 Hz yielded a mean velocity of 0.08 m/s. For trial 2, *f* = 30 Hz yielded 0.22 m/s, and for trial 3 *f* = 40 Hz yielded 0.30 m/s. These mean velocities were chosen to recreate three different scenarios of turbulence and sediment transport, with means speeds fast enough to keep eggs in suspension, but not too fast as to create large bedforms or sheet flow conditions able to sweep away all sediment from the test section. The selected range of velocities is also found in natural streams where grass carp eggs and larvae have been collected (e.g. the Sandusky river [7]), where mean water velocities in some portions of the river rarely exceed 0.3 m/s (USGS gage station 04198008 near Wightmans Grove OH).

Vertical profiles of longitudinal velocity *u* (in the *x*-direction), transverse velocity *v* (in the *y*-direction), and vertical velocity *w* (in the *z*-direction), were measured by recording instantaneous velocities for 3 minutes at each point, at a sampling rate of 100 Hz. Measurements were taken at every millimeter in the *z*-direction along the first centimeter from the sediment bed, and then at every 2 centimeters until the free surface, using an Acoustic Doppler velocimeter (ADV, Nortek-Vectrino). Given the configuration of the ADV, no measurements were taken within the top 5 cm of the water column. From these ADV measurements, two dimensionless numbers (Reynolds (*Re*) and Froude (*Fr*) numbers) were estimated to characterize the turbulence (*Re* > 4x10^3^ fully turbulent flow) and to readily identify subcritical (*Fr* < 1) and supercritical flows (*Fr* > 1) for each condition. Reynolds numbers are computed as *Re* = *UR*_*h*_/*ν*, where *R*_*h*_ is the hydraulic radius. And Froude numbers are computed as $Fr=U/\sqrt{gH}$. Turbulence statistics were calculated using Reynolds decomposition to obtain velocity fluctuations for each velocity component (*u*′,*v*′,*w*′), as:

$$u^{\prime }=u-U$$ (4A)

$$v^{\prime }=v-V$$ (4B)

$$w^{\prime }=w-W$$ (4C)

where *u*, *v*, *w* are the instantaneous flow velocities and *U*, *V*, *W* are the time averaged velocities. To evaluate turbulent statistics, Reynolds stresses, (〈*u*′*v*′〉, 〈*u*′*w*′〉, 〈*v*′*w*′〉, indicators of fluxes of momentum in the flow), turbulence intensities, (*u*_*rms*_, *v*_*rms*_, *w*_*rms*_), and turbulent kinetic energy, *k*, are thus calculated as:

$$u_{rms}^{}=\sqrt{\bar{u^{\prime 2}}}$$ (5A)

$$v_{rms}^{}=\sqrt{\bar{v^{\prime 2}}}$$ (5B)

$$w_{rms}^{}=\sqrt{\bar{w^{\prime 2}}}$$ (5C)

$$k=0.5(\bar{u^{\prime 2}}+\bar{v^{\prime 2}}+\bar{w^{\prime 2}})$$ (6)

Friction velocity, $u_{*}=\sqrt{\tau_{b}/\nu }$, with *τ*_*b*_ as the bed shear stress, was calculated using two methods:

1. Fitting the measured velocity profile to the law of the wall [28]:
  $$u^{+}=\frac{1}{K}\ln\left(z^{+}\right)+5.1$$ (7)
  where *u*^+^ = *u*/u_*_, *z*^+^ = u_*_z/*ν*, *z* is the vertical distance from the bed, and *κ* = 0.41 is the von Karman constant [29].
2. Using a turbulent kinetic energy approach, estimating shear stress as [30–31]:
  $$\tau_{b}=C_{1}\rho [0.5(\bar{u^{\prime 2}}+\bar{v^{\prime 2}}+\bar{w^{\prime 2}})]$$ (8)
  where *C*_*1*_ = 0.19 [32].

Different criteria exist to analyze the expected behavior of particles (eggs and larvae in our study) in suspension [33–34]. We compared the settling velocity of the particle with the friction velocity and the vertical turbulent intensity, assuming that vertical fluctuations generated by the bed shear stress will overcome the settling speed to maintain the particles in suspension [33]. Dimensionless ratios between vertical turbulent intensities, friction velocities, and settling velocity, were calculated to better explore the variable space regarding critical resuspension parameters. A suspension parameter, evaluated as the inverse of the Rouse number, *Z*_*R*_, [33] was calculated as:

$$\frac{1}{Z_{R}}=\frac{\beta \kappa u_{*}}{W_{s}}$$ (9)

where $\beta =1+2{\left(\frac{W_{s}}{u_{*}}\right)}^{2}$ is a coefficient related to diffusion of sediment particles, with a maximum value of *β* = 3.0. Following Hearn’s criteria (2008) [34] for sediment particles in suspension as bedload (*1/Z*_*R*_ <0.4), approximately 50% suspended sediment (0.4<*1/Z*_*R*_ <0.8), approximately 100% suspended sediment (0.8<*1/Z*_*R*_ <1.3), or wash load (*1/Z*_*R*_ >1.3), we classified the eggs as suspended particles using the inverse of *Z*_*R*_.

#### Survival rates in RTF

On day 5 of each trial, surviving larvae were counted to determine survival rates. A water bath with similar water temperature as in the RTF (23–24°C) was used as a baseline to ascertain survival differences with the RTF. The water bath (1.8 m *x* 0.9 m) housed 4 aquaria (0.3 m *x* 0.6 m *x* 0.3 m each) with hatching jars where a subset of eggs was kept under ideal oxygenation conditions and gentle upwelling flow to prevent external damage. Each aquarium kept 500 eggs during trial 1 and trial 3; but only 100 during trial 2. Water temperature in the water bath and RTF was monitored with HOBO temperature loggers (Onset Computer Corp) recording at 15-min intervals. A 500-gallon tank, refilled in every trial with well water from the same source, supplied the water bath and the flume throughout the experiments.

## Results and discussion

### Settling column test

**Fig 2:**
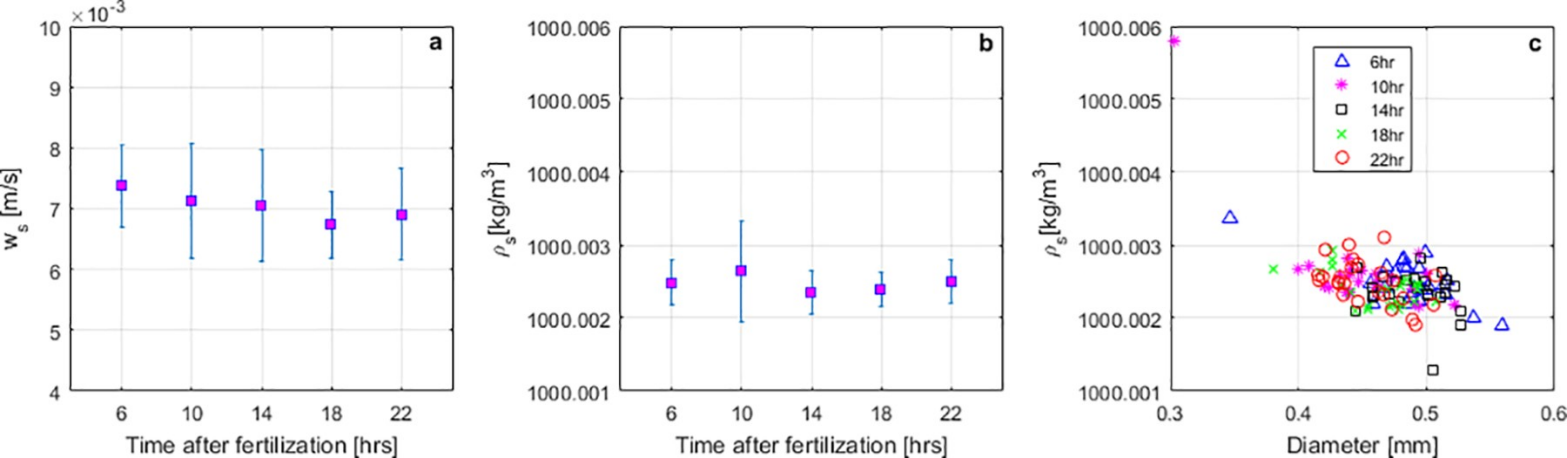
Settling column results for all 4-hour intervals after fertilization. (a) Error bars for egg settling velocity. (b) Error bars for egg density. (c) Comparison between egg diameter and egg density for all samples at each time interval.

Error bars were used to present the results for the settling velocities (*w*_*s*_) and densities (*ρ*_*s*_) of eggs at 6, 10, 14, 18, and 22 hours after fertilization (Fig 2A and 2B). These plots show almost no change between mean settling velocities and mean densities (middle squares) as the fish develops inside the membrane. Mean settling velocities of 7*x*10^-3^ ± 3.5*x*10^-4^ m/s with standard deviations within each batch of {5.5–9.4}x10^-4^ m/s were estimated. The ANOVA test revealed a p-value of 0.0538 (>0.05), which confirms that no significant difference exists between the mean settling velocities of water-hardened grass carp eggs at post-fertilization time. This result is consistent with previous experiments reporting a constant terminal fall velocity of 7.4*x*10^-3^ ± 1.6 *x*10^-3^ m/s [22].

Mean densities of 1000.0025 ± 1.45*x*10^-4^ kg/m^3^ with standard deviations within each batch of {2.32–6.84}x10^-4^ kg/m^3^ were estimated. This is a very narrow range of mean densities considering the wide range of diameter sizes (3–6 mm) (Fig 2C), which also falls within the constant range estimated by George et al. [22] (999.87 ± 1.2 kg/m^3^). The ANOVA test revealed in this case a p-value of 0.1019 (>0.05), which also confirms that no significant difference exists between the mean densities. The estimated density proves that grass carp eggs can be heavy enough to sink in still water but light enough to remain in suspension at low flow velocities, thus avoiding burial and abrasion with bed material. In addition, as discussed by George et al. [22] this strategy provides for reduced mortality resulting from both sight-feeding and filter-feeding predators, and distributes the eggs and larvae broadly within the water column, rather than creating easily-attacked concentrations at the sediment-water or air-water boundaries.

### Race-track flume test

#### Flow characterization

**Fig 3:**
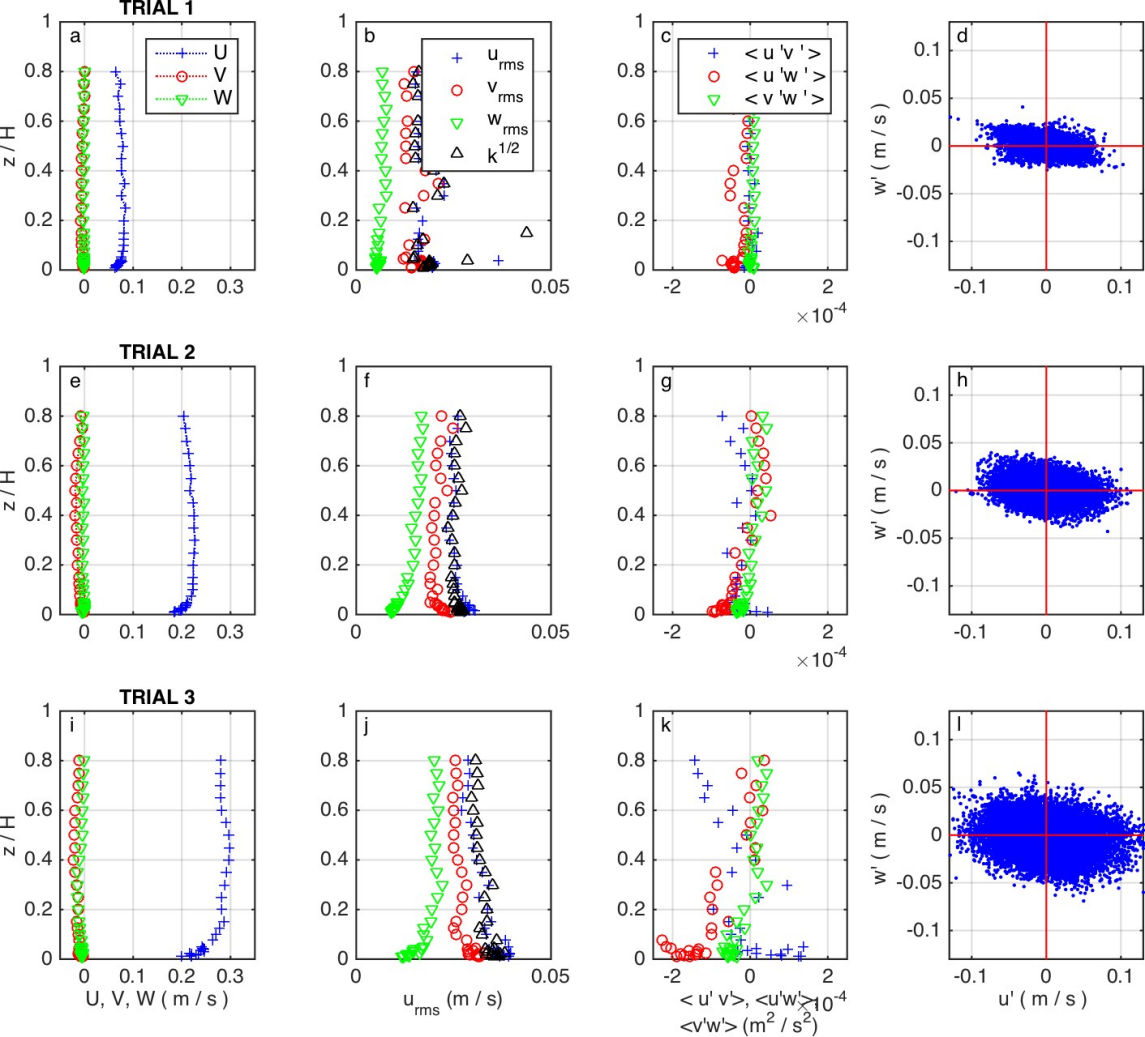
Flow and turbulence conditions over the flume’s straight test section in each trial. (a,e,i) Mean velocities *U*, *V*, *W*. (b,f,j) turbulence intensities *u*_*rms*_, *v*_*rms*_, *w*_*rms*_ and turbulent kinetic energy *k*. (c,g,k) Reynolds stresses 〈*u*′*v*′〉, 〈*u*′*w*′〉, 〈*v*′*w*′〉. (d,h,l) Quadrant analysis from instantaneous velocity measurements 1 mm above the bed.

Reynolds numbers of *Re* = {5–19}x10^3^ and Froude numbers of *Fr* = {0.04–0.15} were computed for the three mean velocities. These values indicate that all trials had fully turbulent and subcritical flow conditions. Vertical profiles of time averaged velocities are shown in Fig 3A, 3E and 3I. Data show low values of both *V* and *W*, below 5% of the longitudinal speed *U*. Fig 3 also shows turbulence intensity and turbulent kinetic energy, *k*, increasing with increasing mean velocity. Profiles of *k* (Fig 3B, 3F and 3G) show near-uniform values through the water column except for the expected peak near the bed. Reynolds stresses profiles show the expected large near-bed values of 〈*u*′*w*′〉 (Fig 3C, 3G and 3K).

Friction velocity values were calculated as u_*_ = {0.47,1.17,1.39} × 10^−2^ m/s and {0.43,1.19,1.50} × 10^−2^ m/s, using log-law (Eq 7) and turbulent kinetic (Eq 8) approaches for the three mean flows studied, respectively, showing a good agreement between both methods (variation from 2% to 9%). Instantaneous turbulent fluctuations (*u’*,*v’*,*w’*) can be used to identify the presence of instantaneous events that may influence the resuspension of eggs and larvae and their interaction with the sediment bed. We identified a large proportion of instantaneous events where the instantaneous longitudinal velocity, *u*, fell below the mean value, whereas the instantaneous vertical velocity, *w*, was above the mean vertical component, leading to parcels of fluid moving slower than the mean flow (i.e., *u'* (*= u–U*) *< 0*) experiencing an upwards displacement (i.e., *w'* (*= w–W*) > 0), an event commonly known as 'ejections'. Another large proportion of events is represented by faster moving parcels displacing downwards (*u’ > 0*, *w’ < 0*), which are commonly known as 'sweeps'. Dominance of near-bed sweeps and ejections (Fig 3D, 3H and 3L) indicates a high frequency of events able to lift or re-suspend material from the bed into the water column. For eggs, with a density slightly above that of water, these events are sufficient to keep them in suspension even at the lowest velocities, as seen in previous studies (e.g. Garcia et al. [18]). For larvae, we also expect that these coherent flow structures help them stay suspended during period 2 when the larvae swim only vertically. However, once they reach period 3, they have the ability to swim and remain over less turbulent regions.

**Fig 4:**
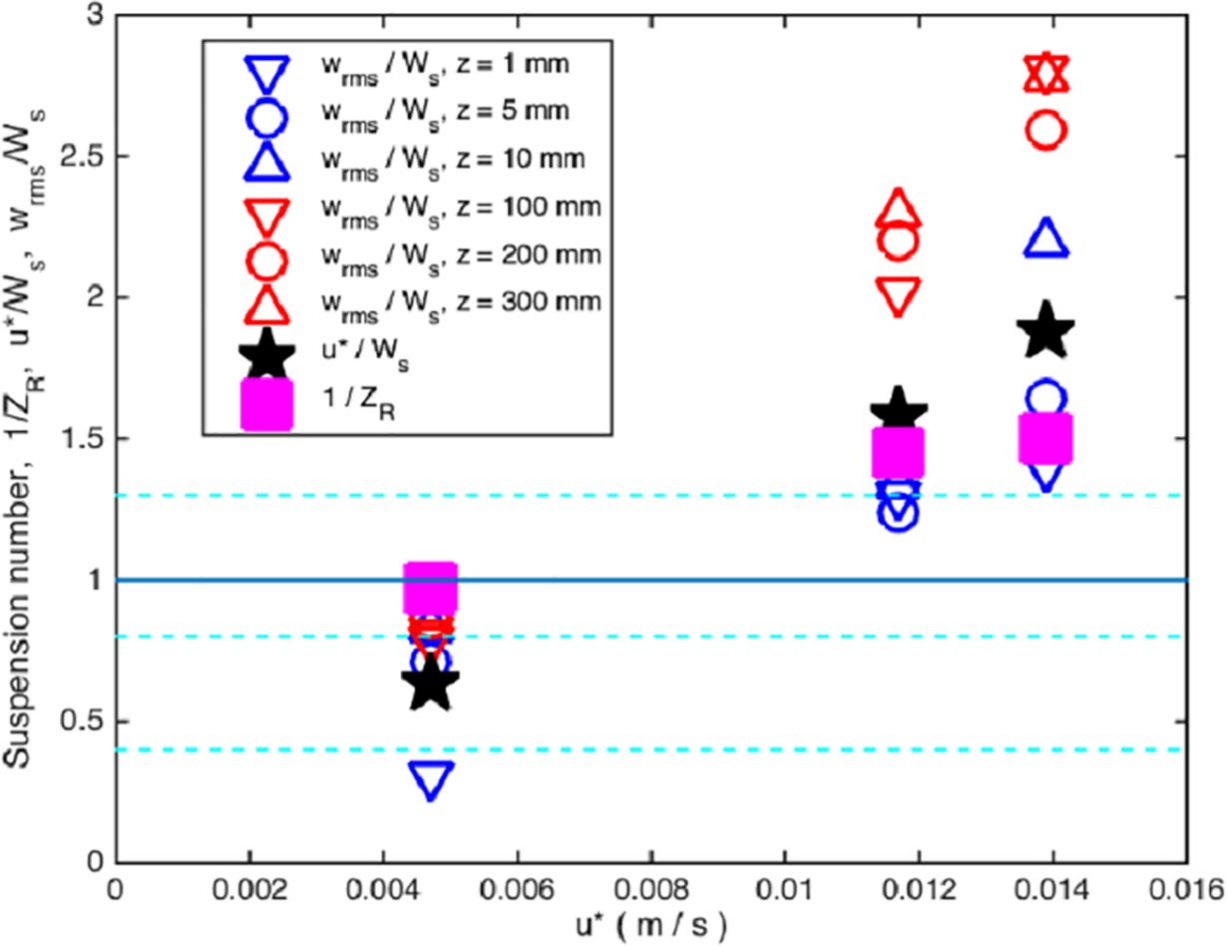
Suspension parameter $\frac{1}{Z_{R}}$, and ratios $\frac{w_{rms}}{W_{s}}$ and $\frac{u_{*}}{W_{s}}$ to classify eggs as suspended particles according to sediment transport criteria. Dashed lines mark delimiting values of the inverse of the Rouse number for bed load (*1/Z*_*R*_ <0.4), approximately 50% of suspended sediment (0.4<*1/Z*_*R*_ <0.8), approximately 100% of suspended sediment (0.8<*1/Z*_*R*_ <1.3), and wash load (*1/Z*_*R*_ >1.3).

Moreover, we classified the eggs as suspended particles using the inverse of *Z*_*R*_. Fig 4 shows calculated values of 1/*Z*_*R*_, as well as the ratios *w*_*rms*_/*W*_*s*_ measured at several water depths, and the ratio friction velocity to settling speed u_*_/*W*_*s*_. Notice the agreement with Hearn’s criteria, with an expected 100% of particles in suspension even at the lowest speed.

### Survival rates

Survival rates in the hatching jars for each trial were 53% in trial 1, 4.5% in trial 2, and 75% in trial 3; whereas in the RTF, where similar temperature with the hatching jars was maintained, were 51% (-3.8% with respect to the hatching jars) in trial 1 (low flow speed), 0.3% (-93.3% with respect to the hatching jars) in trial 2 (medium flow speed but low quality eggs), and 8% (-89.3% with respect to the hatching jars) in trial 3 (high flow speed), as shown in Table 1. Mortality for trial 1 in the RTF, with the lower flow speed (0.08 m/s), was not substantially different than the control jars, suggesting that there was very little impact of the physical processes on the eggs themselves at that speed. The eggs had optimal conditions for suspension (i.e., low turbulence levels, low shear levels, and fewer interactions and collisions with fast-moving sediment particles in suspension), avoiding abrasion or burial. Observations and recordings at the disk pump revealed that the shear generated by the rotating disks created a higher speed at the centermost region within pairs of disks. This velocity gradient pushed the eggs towards the center within parallel disks, avoiding collision with the disks themselves (S1 Videos in Supporting Information). The use of this flume, driven by a vertical axis disk pump, minimizes damage to the eggs and larvae due to the propulsion system in comparison to centrifugal pumps.

**Table 1:** Survival rates in RTF and hatching jars after experiment.

Trial	Flow velocity [m/s]	Eggs stocked to flume	Survival rate in flume	Eggs stocked to jars	Survival rate in jars	Difference in survival rates between flume and jars
**1**	0.08	4000	51.0%	2000	53.0%	-3.8%
**2**	0.22	1604	0.3%	400	4.5%	-93.3%
**3**	0.30	4000	8.0%	2000	75.0%	-89.3%

Mortality for trial 2, in both hatching jars and RTF, was mainly influenced by the poor quality of the eggs due to the high temperatures at spawning. In this trial, the physical processes of interaction with sediment particles or high turbulence levels were not the main factor driving mortality. For trial 3, at the highest speed considered (0.30 m/s), the combined effect of higher levels of turbulence and shear experienced by eggs and larvae, as well as abrasion by interaction with fast moving sediment particles in suspension, substantially increased mortality in comparison to the hatching jars (-89.3%). In this trial, the mortality rates were thus driven by a combination of these abiotic factors, but the contribution towards increased mortality from each individual process could not be quantified. This will be the focus of a future study, isolating pure turbulence and shear effects.

In this study, we registered some percentage of mortality in all trials in the hatching jars, with a minimum of 25% in trial 3. This confirms that mortality is always high in early life stages of fish [35] and can occur due to purely biological reasons. George et al. [15] suggested that burial could be a major cause of mortality of grass carp at embryonic stages, and that settling can be detrimental to their hatching rates, even if they remain uncovered.

### Vertical distribution of eggs and larvae

**Fig 5:**
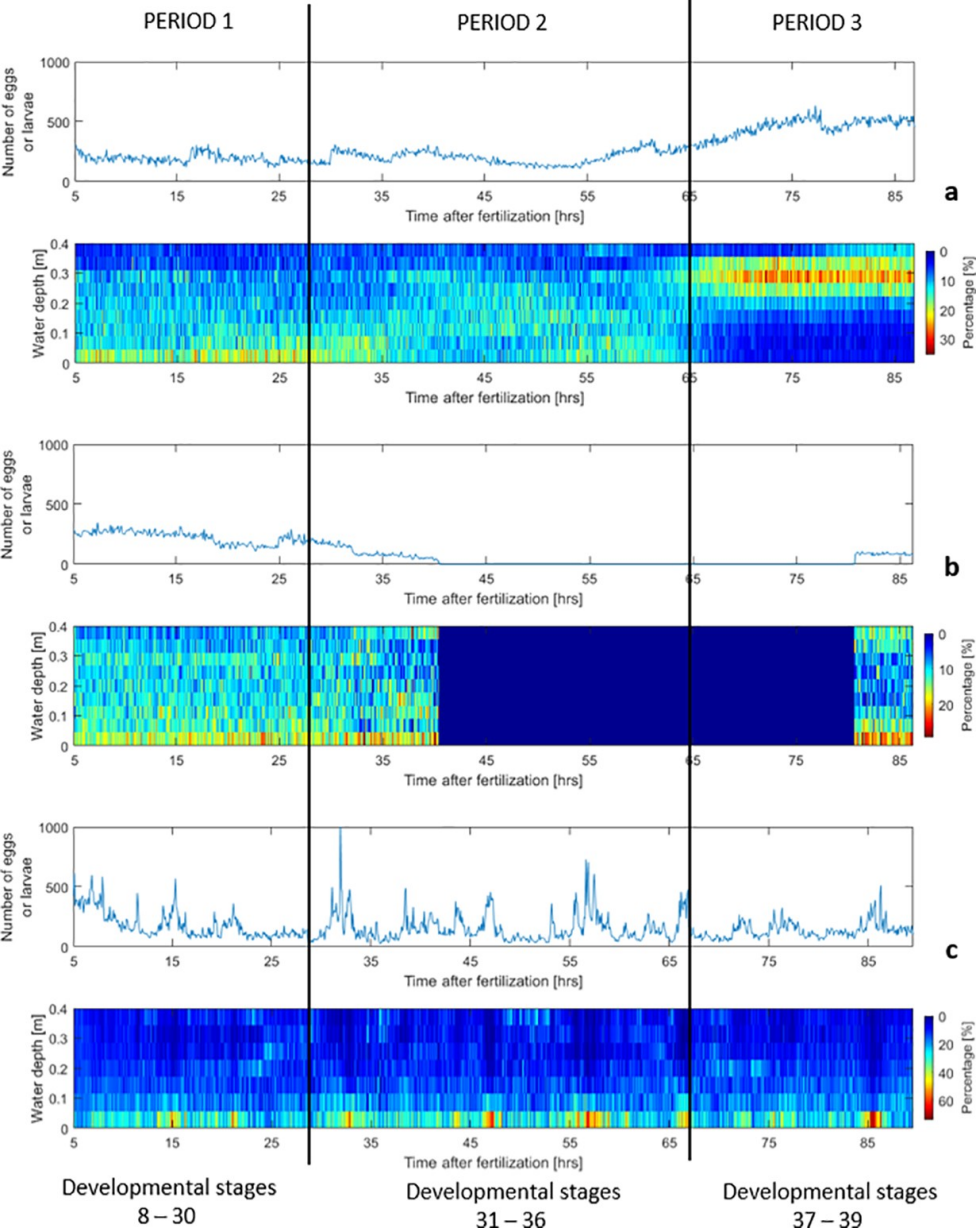
Vertical distribution of egg/larvae across the water depth and number of particles identified along the length of the experiments. (a) trial 1 (0.08 m/s), (b) trial 2 (0.22 m/s), (c) trial 3 (0.30 m/s).

For trial 1 (Fig 5A), in period 1, eggs were drifting mostly within the lower 75% of the water column, with relatively large abundance near the bed. In the vertical-swimming period 2, larvae were noticeably more uniformly spread throughout the water column. In period 3, larvae swam horizontally and remained suspended below the surface, in the top half of the water column, but slightly away from the free surface. Period 3 larvae showed preferential swimming far from the bed (avoiding higher shear and turbulence), and away from free surface (avoiding potential predators). Field collections of larvae in off-channel low velocity habitats often include period 3 larvae but rarely earlier periods [27], thus selection of low velocity conditions detected in this study may be part of a volitional migration to nursery habitats.

For trial 2 (Fig 5B), in period 1, eggs spread almost uniformly across the water column, with a slightly larger number concentrated near the bottom. In period 2, as the survival rate was very low in the RTF (0.3%, only 5 larvae recovered out of 1,604 eggs), the experiment was stopped 2 hours after the end of period 1. In order to get data for period 3, at the end of trial 3, the surviving larvae in the flume (in stage 39) were tested with the velocity of 0.22 m/s to recreate conditions of trial 2. Under these flow conditions, larvae were able to swim and remain suspended preferentially near the surface and near the bottom, seemingly avoiding areas of higher speeds.

For trial 3 (Fig 5C), the vertical distribution of eggs and larvae was fairly constant throughout the experiment. Eggs and larvae were drifting more towards the bottom where they collided with the sediment particles that were also transported by the flow. Increased sediment motion augmented the number of interactions of eggs and larvae with such rapidly-moving particles, increasing the potential for damage from abrasion, even though the eggs were in suspension. These interactions also affected the dispersal speeds of eggs and larvae across the water depth and their vertical distributions.

The vertical distributions presented in Fig 5 can be considered as a reference for research and management purposes in terms of eggs and larvae collection or sampling on field. Eggs and larvae show a prominent tendency to be located below the first quarter of the water depth, even with the fastest velocity presented, than near the water surface. This may support the fact that in previous field studies, e.g. Embke et al. [7], researchers struggled to collect even a few eggs in what they called “the first direct confirmation of grass spawning in a Great Lakes tributary”. They used paired bongo nets of 0.5 m diameter just below the water surface for an interval of 5 minutes during high-flow events on the Sandusky river. We recommend for future field campaigns the use of nets that allow sampling at lower depths to increase the probabilities of successful collection.

### Traveling and swimming speeds

**Fig 6:**
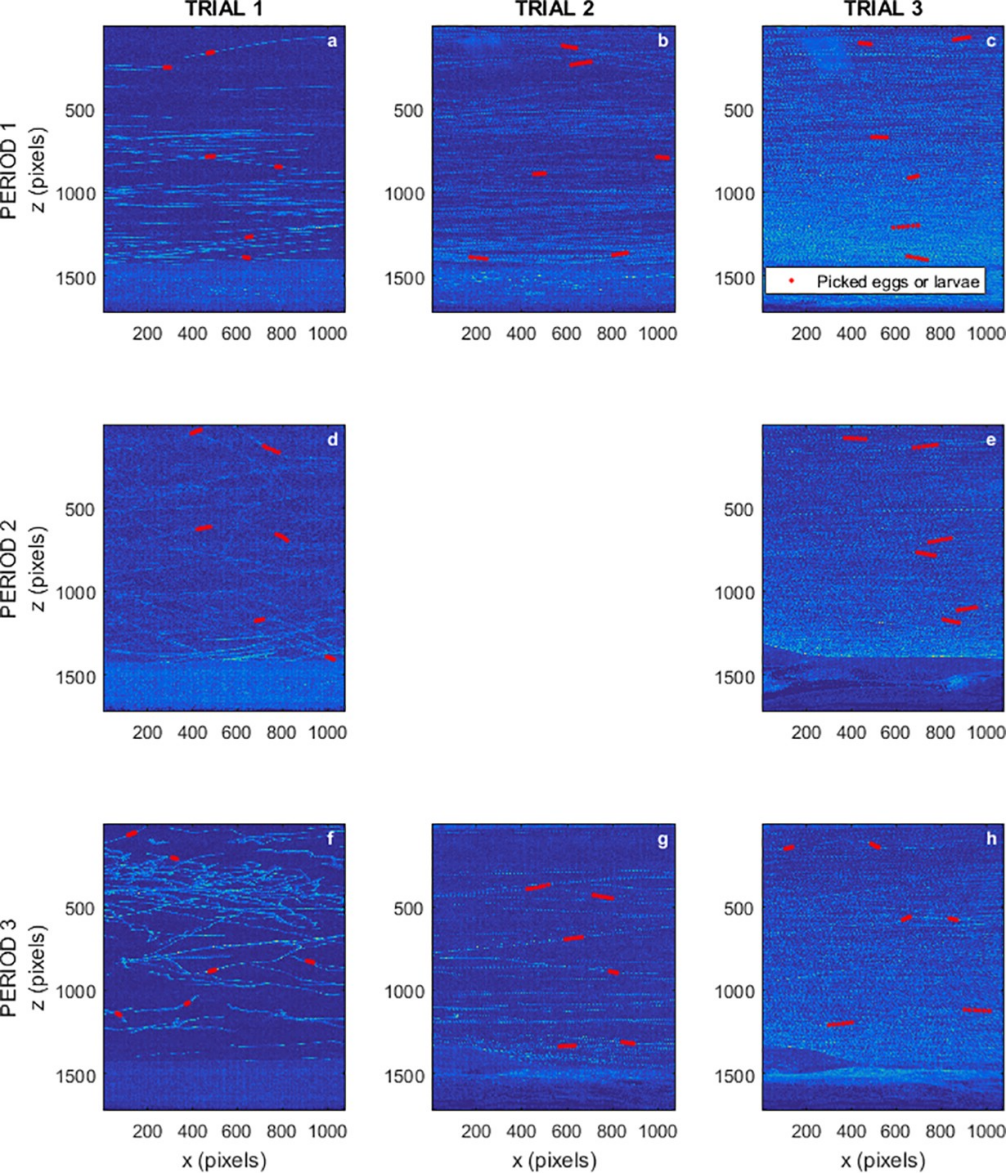
Composite images showing eggs and larvae trajectories at every developmental period and flow speed. (a,d,f) Trial 1. (b,g) Trial 2. (c,e,h) Trial 3. For developmental periods: Period 1 (a-c), Period 2 (d-e), and Period 3 (f-h). Red trajectories shown as examples of those used to estimate traveling and swimming speeds.

Vertical traveling speeds of eggs and larvae were estimated over three regions of the water column: bottom (0 to 0.15 m from the bed), middle (0.15 to 0.30 m), and top (0.30 to 0.40 m) to quantify the effect of turbulence and coherent flow structures on drifting patterns (Table 2). In accordance with previous experiments (e.g. Garcia et al. [18]), we observed trajectories of eggs and larvae with both negative and positive slopes (Fig 6). Eggs moved downwards at speeds of up to 4.2*x*10^-2^ m/s in the middle of the water profile for the mean flow velocity of *U* = 0.22 m/s. This indicates that some of the eggs were not falling downward at their settling speed in the absence of flow (~7*x*10^-3^ m/s) but were being pushed downward by the flow fluctuations instead. Likewise, turbulent forces were also able to cause some proportion of eggs to rise at rates up to 0.048 m/s near the bed for the mean flow velocity of *U* = 0.30 m/s.

**Table 2:** Estimated minimum and maximum vertical traveling speeds, *w*_*t*_, at each of the water profile regions defined. Bottom (0 to 0.15 m above the bed), middle (0.15 to 0.30 m), and top (0.30 to 0.40 m).

			*w*_*t*_ [*x*10^-2^ m/s]		
Period	Developmental stages [27]	*U* [m/s]	BOTTOM	MIDDLE	TOP
**1**	8–30	0.08	-1.16–1.03	-1.25–0.64	-1.64–0.14
		0.22	-2.98–2.35	-1.04–1.18	-4.15–3.45
		0.30	-3.01–4.88	-2.42–0.52	-3.43–1.94
**2**	31–36	0.08	-2.62–4.36	-3.80–10.24	-5.60–10.40
		0.22	–	–	–
		0.30	-3.95–4.03	-5.54–4.25	-3.95–1.46
**3**	37–39	0.08	-2.33–3.76	-2.28–3.12	-4.13–1.84
		0.22	-0.97–2.08	-3.05–1.94	-6.02–4.60
		0.30	-3.64–1.87	-3.92–2.14	-2.42–4.92

Larvae showed the active-passive drift mode described by Zens et al. [36] (also called behavioral drift [37]), in which the larvae did not always actively swim, instead occasionally drifting with the current. The shape of the swimming path for larvae in period 2 for all flow speeds was a negative parabola (which opens downwards), where the focal length and general shape differ according to the speed of the water. Slower water produced a narrower, steep-sided parabola; and faster water produced a wider, more softly curving arc, showing a more active response from the larvae at higher flow speeds, compared to the behavior observed in the absence of flow [16]. Vertical traveling speeds for larvae in period 2 were greater than those of the embryonic period, due to their vertical swimming capabilities; i.e. absolute vertical displacements were faster due to the parabolic self-movement. Conversely, for larvae in period 3, vertical traveling speeds are reduced compared to the period 2 since horizontal swimming is predominant at this stage.

**Fig 7:**
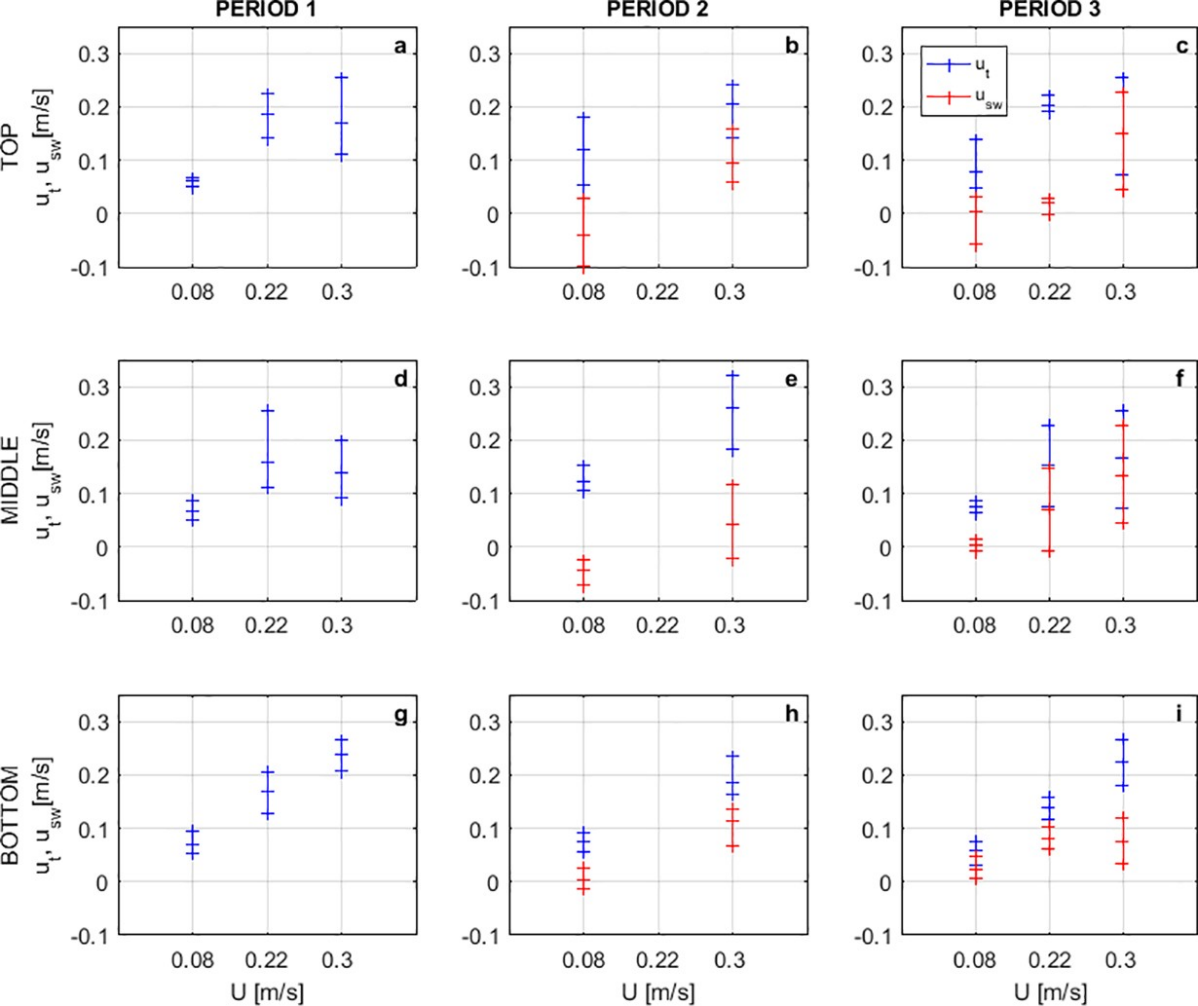
Ranges of horizontal traveling (*u*_*t*_) and swimming speeds (*u*_*sw*_). (a,d,g) Developmental period 1. (b,e,h) Developmental period 2. (c,f,i) Developmental period 3. For three regions considered across the water profile: top (a-c), middle (d-f), and bottom (g-i). Positive traveling speeds in the same direction of the flow. Positive swimming speeds against the direction of the flow.

Horizontal traveling and swimming speeds for eggs and larvae are presented in Fig 7. Eggs were traveling at close to the horizontal mean speed of the flow at any depth, which sounds intuitive, but provides the physical evidence to validate the streamwise drifting of grass carp eggs in numerical models such as FluEgg in its current form.

Larvae were traveling at different speeds than the horizontal mean flow speed, especially in the middle and top regions of the water depth. In period 2, the parabolic swimming trajectory of larvae in the direction of the flow increased also the horizontal traveling speeds. In period 3, larvae swim horizontally facing upstream and offering resistance to the flow. We observed that that resistance was higher as the flow velocity increased since the traveling speeds were very similar for both *U* = 0.22 m/s and *U* = 0.30 m/s, thus there were higher swimming speeds for *U* = 0.30 m/s. The results show that swimming capabilities of the larvae are not negligible and do have a large potential effect on dispersal. Period 2 larvae subsist entirely from endogenous nutrition and do not feed, but period 3 larvae must begin to feed. Rivers where grass carp have been found are typically turbid and carry large quantities of suspended alluvium. Such rivers have little primary productivity and low plankton availability, thus are poor habitats for a larval fish which must feed. In flowing rivers, period 3 larvae likely attempt to move from the river current into low velocity nursery areas [27], and this swimming ability is fundamental in achieving that pivotal change in habitat from flowing river to low velocity feeding and growing areas.

**Fig 8:**
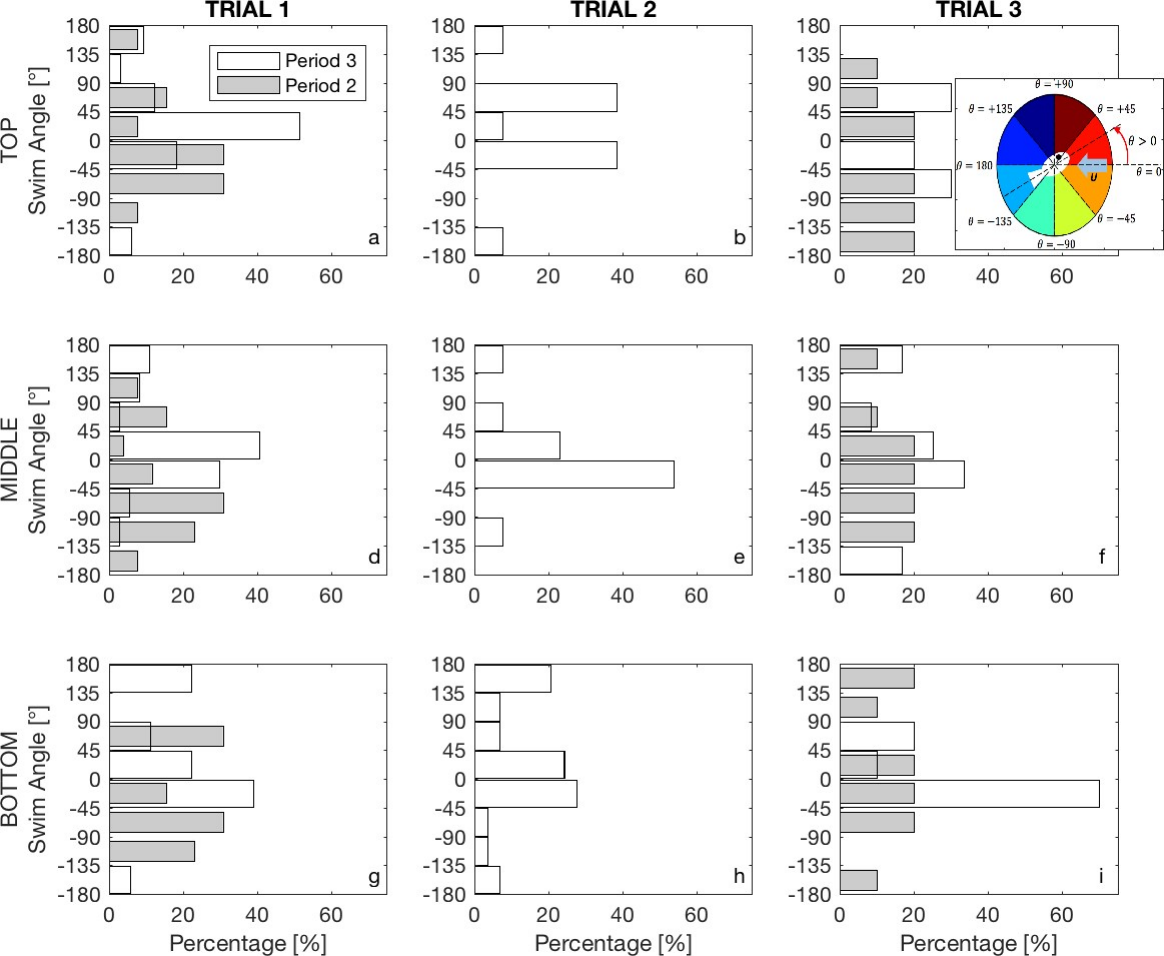
Swimming angle distribution of larvae at periods 2 (grey) and 3 (white). At least 20 larvae per water depth: top (a-c), middle (d-f), and bottom (g-i) were analyzed for each period on trials 1 (a,d,g), 2 (b,e,h), and 3 (c,f,i). Reference frame for swimming angle is indicated in the inset on *c*.

Fig 8 shows the orientation of the larvae for all flow speeds at developmental periods 2 and 3, classified by their location in the water column. The inset in Fig 8C shows the orientation reference frame, with *θ* = 0 degrees horizontally facing (opposing) the mean flow, and data divided in 45 degrees' ranges, positive in counter-clockwise direction. For the lowest mean flow (Fig 8A, 8D and 8G), we notice clear differences between developmental periods, with most larvae able to face the flow in period 2, tending to orient downwards in the bottom and middle sections of the water column, potentially in response to coherent flow structures generated at the bed pushing them towards the surface, and a clear shift once they reach period 3, with improved horizontal swimming abilities to face the oncoming flow within ±45 degrees. For the fastest flow (Fig 8C, 8F and 8I), there is a more uniform distribution during period 2, indicating a reduced ability of larvae to align themselves with the elevated flow speeds, and a clear shift as they reach period 3, now being able to withstand the current and align opposing the mean flow.

**Fig 9:**
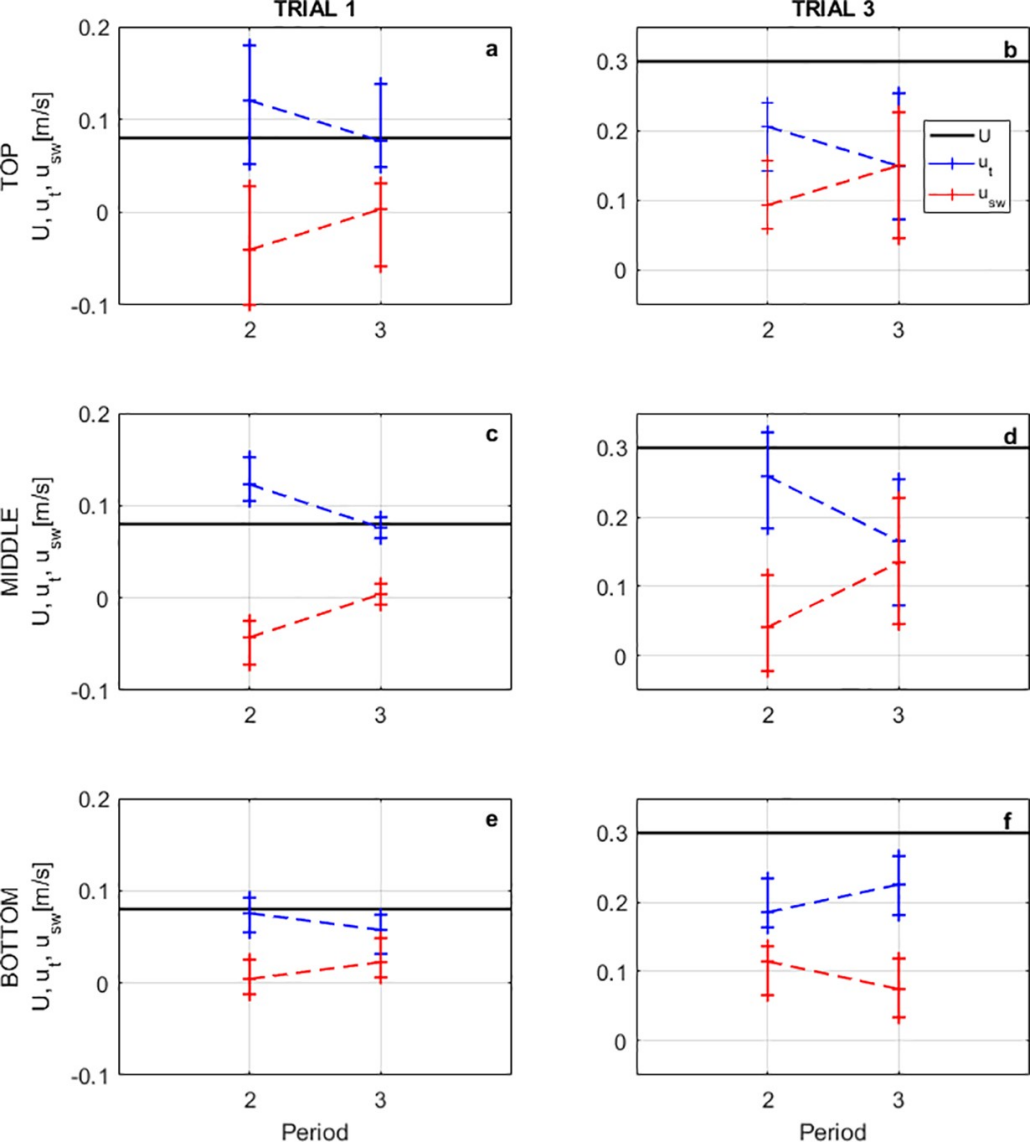
Variation of larvae traveling and swimming speeds between periods 2 and 3 analyzed at each of the three water profile regions. (a,c,e) Trial 1. (b,d,f) Trial 3. For each region in water profile: top (a,b), middle (c,d), and bottom (e,f).

Mean values from Fig 7 for periods 2 and 3, from trials 1 and 3, were used to generate plots that allowed us to observe the influence of the gas bladder inflation in the horizontal swimming speeds of larvae (Fig 9). In Fig 9, we can see that the traveling speeds are reduced after the gas bladder emergence, and swimming speeds are increased; except near the bed for the case of the higher flow (Fig 9F). In this region, higher turbulent intensities and Reynolds stresses are present as shown in Fig 3H and 3I and larvae showed preferences of swimming with the flow, increasing the traveling speed, instead of resisting it as in the middle or top of the water profile.

The swimming speeds opposing the flow direction in period 3 were never higher than the flow velocity, thus larvae always traveled in the same direction of the flow. This pattern was observed in all trials, even for the slowest flow velocity. Larvae adjusted their resistance to the flow proportionally to the drag forces exerted, but did not overcome the flow velocity to swim upstream. These findings provide physical evidence that will allow expansion of the capabilities of numerical tools (e.g. FluEgg [19]) towards predicting the transport of grass carp larvae, with larvae seemingly unable to swim upstream at the earliest life stages.

The observed swimming speeds can be used to estimate larval response once they are able to swim laterally towards the river banks or tributaries looking for nursery habitats, escaping from recirculation zones or areas of high turbulence levels. Because the early life stages of this species represent an important population bottleneck for survival and dispersal, the observed response to various flow conditions is paramount if sampling or collection campaigns are planned, or if management strategies are designed to either reduce the propagation of grass carp North America, or enhance the grass carp recruitment as is normally desired in Asia.

## Conclusions

Grass carp eggs and larvae were used to characterize the buoyancy of eggs, evaluate their drifting and swimming behavior in moving water, and estimate survival rates after their interaction with flow and sediment. Constant ranges of settling velocity and density of post-water-hardened eggs were found throughout the embryonic development (period 1) and were estimated as 7*x*10^-3^ ± 3.5*x*10^-4^ m/s and 1000.0025 ± 1.45*x*10^-4^ kg/m^3^ respectively. This range of density makes the grass carp eggs heavy enough to settle in stagnant waters but light enough to drift even in gentle current. This property minimizes predation and damage that might result from contact with bed material.

Eggs (Period 1) were mainly distributed within the lower 75% of the water column for the three flow velocities analyzed, with a slight tendency to be more concentrated near the bottom. Larvae in period 2, against the slowest flow condition, were more evenly distributed throughout the water column, drifting with the flow on inclined trajectories of slopes of up to 78%. Larvae in period 3 had the ability to remain suspended (swimming) at preferential depths and offer resistance to the flow, with swimming speeds proportional to the flow velocity. This ability allows them to select nursery habitats on river banks or floodplain tributaries, escaping from recirculation zones or areas of higher turbulence levels. Similar depth distribution patterns were not found for the highest flow speed, in which the larvae have a decreased ability to withstand the flow and could not concentrate in preferential depths due to the increased drag exerted on them by the flow, and the increased frequency and intensity of turbulent events and coherent flow structures resulting from the enhanced turbulence field.

The highest velocity case also resulted in high suspended sediment concentrations in the water column, complicating visual identification of eggs and larvae traveling with large sediment particles, and affecting the dispersal speeds and the vertical distributions over time. A clear difference in vertical distribution of the eggs and larvae is found between trials 2 and 3, as mean velocity increases from *U* = 0.22 to 0.30 m/s and u_*_ goes from u_*_ = 1.17 × 10^−2^ to 1.39 × 10^−2^ m/s. This represents a change in the capability of the flow to keep particles in suspension, with both sediment and eggs and larvae facing stronger vertical velocity fluctuations for the 0.30 m/s case.

Temperatures at spawning directly influenced the quality of eggs and consequently the survival rates in hatching jars and in the RTF. Using control batches of eggs maintained under ideal oxygen and temperature conditions in hatching jars as baseline (no damage due to interaction with sediment) we compared survival rates with those found under different flow conditions. The slowest flow studied, still energetic enough to keep eggs in suspension, yielded a survival rate similar to the hatching jars (only -3.8% in percentage difference with respect to the jars), showing optimal conditions avoiding burial and abrasion of eggs rolling in the bed, with minimal interactions with suspended sediment particles and low turbulence levels. The fastest flow, however, showed a significant increase in mortality, -89.3%. We attribute increased mortality to a combination of higher shear and turbulence experienced by the eggs and larvae, and abrasion by fast moving suspended sediment particles, although relative contributions of each process are still unknown.

These findings, along with the drifting and swimming speeds estimated, can be used to identify favorable hydraulic conditions for grass carp spawning and hatching, as well as to estimate more realistic dispersion coefficients to be applied in numerical models that simulate the fate and transport of grass carp eggs. The findings are applicable in North America, where successful reproduction by wild grass carp is generally considered problematic, and in Asia and other locations where enhancement of grass carp recruitment is considered beneficial. The decrease in survival resulting from higher turbulence is especially interesting because it might be useful in devising invasive fish control mechanisms that are based on increased turbulence which might be created by weirs or similar devices that are deployed during the carp spawning season. Changes in river morphology that remove such excess turbulence might be considered in areas where grass carp enhancement is desirable.

While further work is needed to investigate the influence of more realistic scenarios regarding bed morphology and the impact of turbulence levels in mortality, the interactions described by this study between the turbulent structures, eggs or larvae, and sediment, will allow us to reformulate prediction models (e.g. FluEgg, [19]), complement them with estimates of survival rates to expand simulation beyond the hatching stage, improve dispersal estimates, and introduce more realistic boundary conditions. While all of these measurements were performed on grass carp, grass carp have early life stages and behavior that are broadly similar to some other carps native to Asia, notably the bigheaded carps, which have achieved extremely high and problematic populations in the large rivers of the central United States. Thus, these results may be transferrable to those other species.

## Supporting information

S1 Videos Recordings made at the disk pump during Trials 1–3 for eggs stage. (ZIP)
